# Medical Notes and Reflections

**Published:** 1857-04

**Authors:** 


ART. VI.—*Medical Notes and Reflections.* By SIR HENRY HOLLAND, Bart., M. D., F. R. S., etc. etc. From the Third London Edition. BLANCHARD AND LEA, Philadelphia. 1857.

The first edition of these Notes and Reflections was given to the public in 1839, and was republished in this country during the same year. The work has passed to its third edition in England, and from which the volume before us has been issued by the enterprising Philadelphia publishers.

In a brief preface to this third American edition, the author pays a handsome compliment to the profession of our country, and expresses himself as earnestly desirous of gaining a name and good opinion amongst them.

This edition differs in many particulars from the previous ones. Some chapters have been omitted and their places supplied by other subjects. The experience of the past sixteen years has been rendered available in perfecting the subjects treated of in the work.

As the title indicates, the volume is composed of papers on various subjects, being the results of the practical experience, or the reflections of the author, such as might be expected to emanate from an intelligent, thoughtful man, who has noted down through a long series of years what interested his mind, and had been made the subject of observation and study. That they should have passed through successive editions is evidence that they have found favor with the profession. That the number has not been greater, is evidence, also, that they possess nothing meretricious, such as would dazzle by a false brilliancy, or arrest attention by the boldness of noval theories.

Indeed, they are such papers as would be read rather by the established practitioner, than by the student pressing on with all his vigor for the day of release from his allotted term of pupilage, and who will enter the profession with a certainty of the positive character of his knowledge of all that appertains to the science of medicine. The work partakes rather of the character of the quiet conversational recapitulation of medical experience, and perhaps, disappointments too, which one medical man would indulge with another, in the moments of social intercourse.

It is true, that these characteristics are the ones which should commend themselves to those preparing for the discharge of their professional duties, and he will do a kindness to the student who will point out to him such a course of reading, or train of thought, as will serve to temper his self-confidence, and warn him that disease is not such a positive character, or rather does not manifest itself with such perfect individuality of character, that merely to see the patient, is to diagnose the disease, and that this made out, remedies can be prescribed by rule, with undeviating certainty of result. The student, as well as the practitioner, can read this volume with profit.

We have not time, or space, for a particular reference to all the subjects of this volume. We can attempt to do nothing more than to express our general appreciation of its meritorious character. It being not a new claimant for public favor, furthermore renders this only necessary. N.

